# The expression and evolution of virulence in multiple infections: the role of specificity, relative virulence and relative dose

**DOI:** 10.1186/1471-2148-13-97

**Published:** 2013-05-03

**Authors:** Frida Ben-Ami, Jarkko Routtu

**Affiliations:** 1 Department of Zoology, George S. Wise Faculty of Life Sciences, Tel Aviv University, Tel Aviv, 6997801, Israel

## Abstract

**Background:**

Multiple infections of the same host by different strains of the same microparasite species are believed to play a crucial role during the evolution of parasite virulence. We investigated the role of specificity, relative virulence and relative dose in determining the competitive outcome of multiple infections in the *Daphnia magna*-*Pasteuria ramosa* host-parasite system.

**Results:**

We found that infections by *P. ramosa* clones (single genotype) were less virulent and produced more spores than infections by *P. ramosa* isolates (possibly containing multiple genotypes). We also found that two similarly virulent isolates of *P. ramosa* differed considerably in their within-host competitiveness and their effects on host offspring production when faced with coinfecting *P. ramosa* isolates and clones. Although the relative virulence of a *P. ramosa* isolate/clone appears to be a good indicator of its competitiveness during multiple infections, the relative dose may alter the competitive outcome. Moreover, spore counts on day 20 post-infection indicate that the competitive outcome is largely decided early in the parasite’s growth phase, possibly mediated by direct interference or apparent competition.

**Conclusions:**

Our results emphasize the importance of epidemiology as well as of various parasite traits in determining the outcome of within-host competition. Incorporating realistic epidemiological and ecological conditions when testing theoretical models of multiple infections, as well as using a wider range of host and parasite genotypes, will enable us to better understand the course of virulence evolution.

## Background

In nature, free-living organisms are regularly found to be infected by an assemblage of different parasite species or genetically distinct parasite strains (reviewed in [1,2]). In fact, multiple infections are the norm rather than the exception in diverse host-parasite systems, e.g., anther-smut disease [3], malaria *Plasmodium* spp. [4], insect nucleopolyhedrovirus [5], fungus gardens of the leaf-cutter ants [6]. Coinfections also have implications for human health [7]. Theory has emphasized the importance of multiple infections in a variety of evolutionary processes such as the emergence of resistance to drugs [8], the evolution of sex in hosts coevolving with multiple parasites [9] and particularly the evolution of higher levels of virulence [10-13]. In the absence of spiteful interactions among parasite strains [14] or when the reproductive or exploitative rate of an individual parasite is not limited by the collective action of the coinfecting group [15], most experimental studies suggest that the overall expression of virulence of multiple infections is either higher than the virulence of any of the coinfecting strains as measured in single infections [16-18], or at least as high as the most virulent strain [19-22]. Understanding the determinants of intra-host competition and predicting the course of virulence evolution are thus of outmost importance for public health, medicine and agriculture [23].

The competitive outcome of multiple infections appears to be driven by several interrelated factors: the relative virulence of the coinfecting strains as measured in single infections [19,24], prior residency of one of the parasite strains or species [25-28], and the infectious dose used during simultaneous exposure [18,29,30]. The latter factor – infectious dose – is strongly tied to the parasites’ epidemiology. For example, variation in the infectious dose (i.e., the number of parasite spores a host is challenged with) is known to affect the probability of infection in single [31-33] and multiple infections [34]. Furthermore, the rate at which transmission stages are produced within the host could be influenced by the relative dose of its coinfecting parasites [35,36]. This is important, because all else being equal, a parasite strain that produces more transmission stages will have a greater representation in subsequent infectious doses. Since virulence is proposed to be traded off against parasite transmission ([37,38], reviewed in [39]), it is crucial to understand how the relative dose of each of the coinfecting parasite strains can impact this trade-off. Nevertheless, few studies examined how the relative parasite dose influences the expression of virulence and the production of transmission stages in the presence of multiple infections [18,30].

Another often overlooked factor that may affect the expression and evolution of virulence is the degree of specificity in host-parasite interactions. In theory, highly specialized parasites can evolve towards high levels of virulence ([40], but see also [41]). For instance, peak parasitaemia (a proxy for virulence) was higher in specialist than in generalist malaria parasites of primates, when confounding life-history traits were controlled [42]. An earlier study of simultaneous and sequential multiple infections of *Daphnia magna* using three isolates of its obligate parasite *Pasteuria ramosa* suggested that the most virulent competitor produced most transmission stages [19]. However, *P. ramosa* clones (including the two clones used in the present study) have recently been shown to exhibit much higher specificity than isolates (clones are a single genotype whereas isolates are parasite samples from infected hosts that may contain multiple genotypes; [43]). In other words, *P. ramosa* clones infect fewer *D. magna* genotypes than *P. ramosa* isolates, and therefore the host genotype range of *P. ramosa* clones is narrower than that of isolates [43].

The present study experimentally investigates multiple infections in *D. magna* using infectious doses containing isolates and clones of *P. ramosa* in equal (50:50) and unequal proportions (90:10 and 10:90). By varying the relative representation of *P. ramosa* isolates/clones in the infectious dose (i.e., varying the specificity of multiple infections) and by comparing virulence, host fitness and parasite fitness in single vs. mixed infection treatments, we aim at (i) exploring how the relative virulence in single infections affects the overall expression of virulence during mixed infections, and (ii) assessing the effects of specificity (*P. ramosa* isolates vs. clones) on intra-host competition. In the following we use the term single infections to refer to infectious doses containing either a single *P. ramosa* clone or a single *P. ramosa* isolate. We use the term mixed infections to refer to infectious doses containing a mixture of a single *P. ramosa* clone and a single *P. ramosa* isolate, or a mixture of two different *P. ramosa* isolates.

## Results

### General effects

Between days 5 and 16 of the experiment, 178 of 1,344 *D. magna* individuals died for unknown reasons (13.2%). Such rates of early host deaths are not unusual [19,27]. None of the control *D. magna* became infected. Controls were excluded from the analyses of infection rates and parasite spore production. Infection rates in all infection treatments were above 90%. The few uninfected *D. magna* in the infection treatments were excluded from all analyses. Time-to-host-death in the control group was on average (± SE) twofold longer than that of all infection treatments combined (112.7 ± 3.7 vs. 54.9 ± 0.4 days, Table 1[A]). Host control animals produced 251.3 ± 8.3 offspring per individual, whereas the average of the pooled infection treatments resulted in only 3.6 ± 0.2 offspring per individual.

**Table 1:** Infection contrasts

		**Time-to-host-death (virulence)**	
**Source**	***df***	***F***	***P***
**Corrected model**	23	40.7	**<0.001**
A. Control vs. infection treatments	1	797.2	**<0.001**
**Infection contrasts**			
B. Mixed infections with P1 and P4	2	0.4	0.68
C: P4-100 vs. P1-10 + P4-90 & P1-50 + P4-50 & P1-90 + P4-10 & P1-100	1	7.6	**=0.006**
D: Mixed infections with P1 and C1	2	0.1	0.94
E: C1-100 vs. P1-10 + C1-90 & P1-50 + C1-50 & P1-90 + C1-10 & P1-100	1	34.7	**<0.001**
F: Mixed infections with P1 and C14	2	0.5	0.62
G: C14-100 vs. P1-10 + C14-90 & P1-50 + C14-50 & P1-90 + C14-10 & P1-100	1	39.9	**<0.001**
H: Mixed infections with P2 and P4	2	1.8	0.16
I: P4-100 vs. P2-10 + P4-90 & P2-50 + P4-50 & P2-90 + P4-10 & P2-100	1	6.7	**=0.01**
J: Mixed infections with P2 and C1	2	15.6	**<0.001**
K: C1-100 vs. P2-10 + C1-90 & P2-50 + C1-50 & P2-90 + C1-10 & P2-100	1	27.8	**<0.001**
L: Mixed infections with P2 and C14	2	6.2	**=0.002**
M: C14-100 vs. P2-10 + C14-90 & P2-50 + C14-50 & P2-90 + C14-10 & P2-100	1	28.7	**<0.001**
**Error**	582		

ANOVA contrasts for time-to-host-death. *F* statistic and significance in contrasts in which the degrees of freedom are greater than one are for the joint contrast. All contrasts are orthogonal. Bold typeface indicates significant effects.

### Virulence, host fitness and parasite fitness in single infections

**Figure 1:**
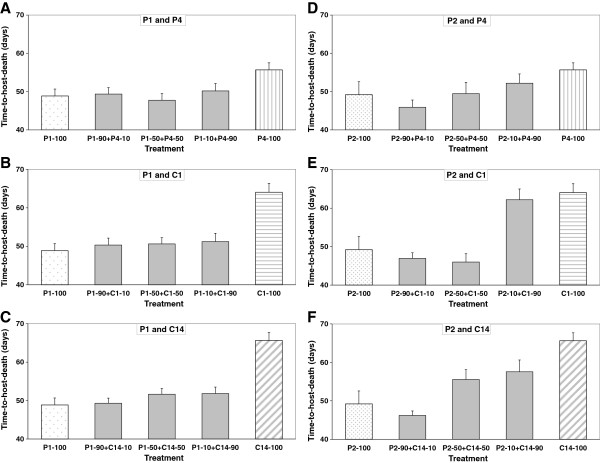
**Time-to-host-death (virulence) in single and mixed infections.** Time-to-host-death (virulence) in single and mixed infections by the parasite isolates/clones **(A)** P1 and P4 **(B)** P1 and C1 **(C)** P1 and C14 **(D)** P2 and P4 **(E)** P2 and C1, and **(F)** P2 and C14. Error bars are standard errors.

**Figure 2:**
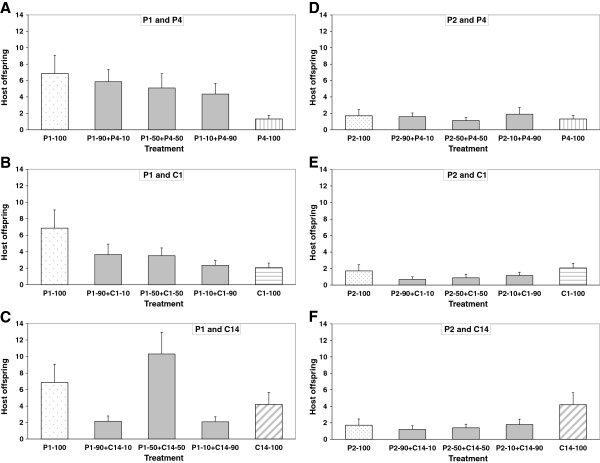
**Lifetime number of host offspring produced (host fitness) in single and mixed infections.** Lifetime number of host offspring produced (host fitness) in single and mixed infections by the parasite isolates/clones **(A)** P1 and P4 **(B)** P1 and C1 **(C)** P1 and C14 **(D)** P2 and P4 **(E)** P2 and C1, and **(F)** P2 and C14. Error bars are standard errors.

We found a significant difference in virulence (defined as time-to-host-death-since-exposure; see Methods further below) among the five parasite isolates/clones, with isolates P1 and P2 being the most virulent irrespective of the infective dose used (Table 2, Figure 1). *Pasteuria ramosa* isolates killed their host faster than *P. ramosa* clones (isolates vs. clones: *F*_1,464_ = 199.6, *P* < 0.001), regardless of dose (isolate/clone * dose: *F*_3,464_ = 0.3, *P* = 0.85). Host offspring production was on average higher in hosts infected by isolate P1 than in hosts infected by the remaining isolates/clones, but it was invariant to dose (Table 2, Figure 2). Spore production differed among the five parasite isolates/clones, but was unaffected by dose (Table 2). *Pasteuria ramosa* isolates produced fewer spores than *P. ramosa* clones, regardless of dose (isolates vs. clones: *F*_1,464_ = 43.3, *P* < 0.001; isolate/clone * dose: *F*_3,464_ = 0.7, *P* = 0.53). The results are similar with and without including the interactions in the model. Since dose level only slightly increased virulence, and even then without interacting with parasite isolate/clone, in the following we only compared the higher dose, single infection treatments (100,000 spores) with multiple infections consisting of spore mixtures where the total amount of spores is 100,000 (i.e., 90,000:10,000, 50,000:50,000 and 10,000:90,000 spores).

**Table 2:** Analysis of variance for host and parasite traits in single infection treatments

		**Time-to-host-death**		**Offspring production**		**Spore production**	
		**(virulence)**		**(host fitness)**		**(parasite fitness)**	
**Source**	***df***	***F***	***P***	***F***	***P***	***F***	***P***
Parasite	4	49.3	**<0.001**	13.5	**<0.001**	12.8	**<0.001**
Dose	3	5.0	**0.02**	2.0	0.16	2.8	0.08
Parasite * Dose	12	1.2	0.27	0.5	0.91	0.9	0.52
**Error**	452						

Two-way ANOVA for time-to-host-death, host offspring production and parasite spore production in single infection treatments. Bold typeface indicates significant effects.

### Virulence in mixed infections

In the case of mixed infections with parasite isolate P1, time-to-host-death was unaffected by the relative proportions of the other isolate or clone (Table 1[B/D/F]). Mixed infections with P1 were as virulent as single infections with P1 (Table 1[C/E/G], Figure 1A-C). In the case of mixed infections with parasite isolate P2, time-to-host-death was only unaffected by the relative proportions of parasite isolate P4 (Table 1[H]). In this case mixed infections with P2 were as virulent as single infections with P2 (Table 1[I], Figure 1D). Although P2 was as virulent as P1 in single infections (49.2 ± 3.4 vs. 48.9 ± 1.8 days), mixed infections with low concentrations of P2 (10%) and high concentrations of parasite clones C1 or C14 (90%) were as virulent as single infections with C1 or C14, respectively. Mixed infections with higher concentrations of P2 (50% or 90%) were as virulent as single infections with P2 (Table 1[J/K/L/M], Figure 1E-F).

### Host fitness in mixed infections

In the case of mixed infections with parasite isolate P1, host offspring production tended to decline with increasing concentrations of P4 and C1 (Figure 2A-B), though this decline was not significant. Mixed infections with P1 and C14 in unequal concentrations (10:90 and 90:10) resulted in fewer offspring than single infections, but in equal concentrations (50:50) they resulted in more offspring than single infections (*F*_2,582_ = 5.8, *P* = 0.003; Figure 2C). In the case of parasite isolate P2, host offspring production did not differ among mixed infection treatments with P4, C1 or C14 (*P* > 0.97; Figure 2D-F). Overall, mixed infection treatments with P1 resulted in the production of significantly more host offspring than with P2 (*F*_1,582_ = 8.7, *P* = 0.003; Figure 2).

### Competitive outcome on day 20 post-infection

**Figure 3:**
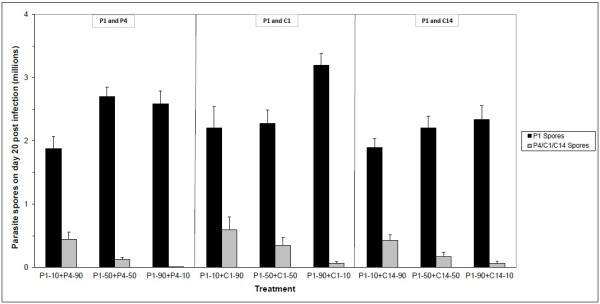
**Parasite spore production on day 20 post-infection in mixed infections.** Parasite spore production on day 20 post-infection by parasite isolates/clones P1 and P4 (left panel), P1 and C1 (middle panel), and P1 and C14 (right panel). Error bars are standard errors.

We used genetic markers to test for the relative success of the competing parasite isolates and clones within individual hosts during the growth phase (day 20) of the disease and upon host death. The superior competitiveness of parasite isolate P1 in comparison with P4, C1 and C14 was largely evident on day 20 post-infection (Figure 3). More precisely, by day 20 isolate P1 produced more spores consistently and increasingly by relative dose: 1.43-1.61 million more spores in concentrations of 10%, 1.93-2.57 million more spores in concentrations of 50%, and 2.27-3.13 million more spores in concentrations of 90% (P1 and P4: F_2,27_ = 10.7, *P* < 0.001; P1 and C1: F_2,25_ = 5.0, *P* = 0.014; P1 and C14: F_2,27_ = 3.5, *P* = 0.043; Figure 3).

### Terminal competitive outcome

**Figure 4:**
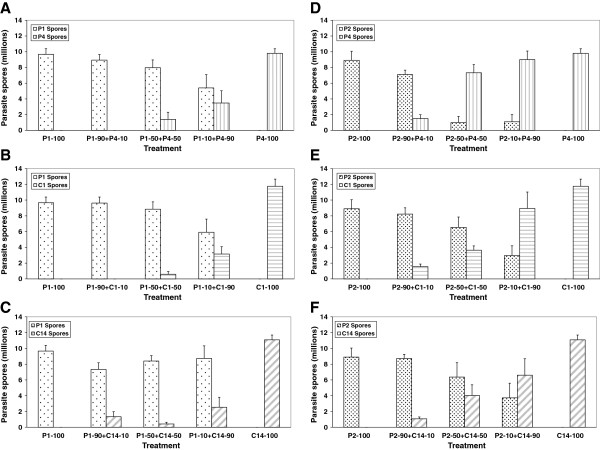
**Lifetime spore production of an infection (parasite fitness) in single and mixed infections.** Lifetime spore production of an infection (parasite fitness) in single and mixed infections by the parasite isolates/clones **(A)** P1 and P4 **(B)** P1 and C1 **(C)** P1 and C14 **(D)** P2 and P4 **(E)** P2 and C1, and **(F)** P2 and C14. Error bars are standard errors.

Mixed infections with parasite isolate P1 in concentrations of 90% or 50% resulted in an almost complete exclusion of P4, C1 and C14 (95% confidence interval for the difference in spore production between P1 and P4/C1/C14 did not include 0; Figure 4A-C). Only when P1 was present in a low starting concentration (10%), both parasites succeeded in producing spores, but both suffered by producing fewer transmission stages than they produced in single infections (Figure 4A-C). In these low concentration treatments, spore production by isolate P1 exceeded that of P4/C1/C14, but the difference was not significant.

Although parasite isolate P2 was as virulent as P1 in single infections, in mixed infections it was less competitive than P1. Only when P2 started with 90%, it mostly excluded the other strains (95% confidence interval for the difference in spore production between P2 and P4/C1/C14 did not include 0; Figure 4D-F). In lower concentrations (i.e., 50% or 10%) isolate P4 nearly excluded P2 (Figure 4D), whereas in the case of P2 vs. C1 or C14, each pair succeeded in producing spores, but both suffered by producing fewer transmission stages than they produced in single infections (Figure 4E-F).

Total spore production (i.e., sum of spores produced by a pair of parasites in mixed infections) did not significantly exceed the amount of spores produced by the parasite that produced more spores in the pair during single infections (Figure 4A-F).

## Discussion

In single infections we found that *P. ramosa* isolates killed their hosts faster and produced fewer spores than *P. ramosa* clones. We also found that two similarly virulent isolates of *P. ramosa* differ considerably in their competitiveness when faced with coinfecting *P. ramosa* isolates and clones. While isolate P1 almost completely prevented the less virulent isolate P4 and the less virulent clones C1 and C14 from producing spores regardless of their relative dose (Figure 4A-C), in the case of isolate P2 the relative dose affected the competitive outcome (Figure 4D-F). Despite P1 being a better competitor, single and mixed infections with P1 resulted in the production of more host offspring than with P2 (Figure 2). Mixed infections were as virulent but not more virulent than single clone infections, and thus neither resulted in overexploitation of the host by the parasites (i.e., time-to-host-death and total spore production were not higher in mixed infections; Figures 1 and 4), nor entailed additional costs upon the host because there was no further reduction in host fecundity (Figure 2). The competitive ability in treatments with equal concentrations (50:50) appears to be transitive, i.e., against both reference isolates P1 and P2, isolate P4 competes better than clone C1 which competes better than clone C14. Although the *P. ramosa* isolate/clone with the higher starting dose has a higher likelihood to succeed, its success ultimately depends on its competitiveness (Figure 4). Based on spore counts 20 days post-infection, it appears that the competitive outcome is largely decided during the first half of the parasite’s growth phase (Figure 3).

Our results extend previous studies of multiple infections in the *D. magna*-*P. ramosa* host-parasite system, which were conducted using just parasite isolates [19,27]. First, we show for the first time that the effects of multiple infections by parasite clones could be different than those previously reported for isolates, because *P. ramosa* clones were less virulent yet produced more transmission stages than *P. ramosa* isolates. Second, we show that *P. ramosa* isolates/clones vary in their within-host competitiveness and ability to induce host castration. Third, we show that epidemiology (i.e., relative dose) affects the outcome of within-host competition (previous studies in this system used the same dose but in equal concentrations). Taken together, these results highlight the need to investigate multiple infections using a wider range of host and parasite genotypes and under diverse epidemiological scenarios.

Parasites that castrate their hosts are expected to inhibit host reproduction early in the infection process, in order to divert host resources for parasitic use [44-46]. The higher fecundity of *D. magna* singly infected with P1 in comparison with other isolates/clones suggests that some *P. ramosa* clones are more successful at inducing castration. If inducing castration bears a cost to the parasite, in the form of slowing down spore development and growth, then it may affect its competitive ability with other clones. In mixed infections, the inability of P1 to castrate its host as quickly as P2 may be compensated by the greater competitiveness of P1. Put differently, castrating the host after it has reproduced once may be less costly to the parasite than doing so immediately after penetration, and may allow the parasite to focus on replicating itself to achieve a competitive edge [47]. P1 might also be benefitting if coinfecting isolates/clones in mixed infection induce castration [48]. It could be argued that *P. ramosa* sterilizes *D. magna* mechanistically, e.g., by growing around its ovaries. This is likely to bear no costs to the parasite, and may be supported by the fact that antibiotic treatment is sufficient to regain host reproduction [49]. However, our day 20 post-infection data suggest that P1 grows faster than its competitors, despite delaying castration. Furthermore, it is not unusual for infected *D. magna* to release a clutch after a long period of castration.

The transitive relationship in competitiveness in mixed infections with equal concentrations (i.e., spore production of P4 > C1 > C14) is in line with their relative virulence in single infections (P4 was more virulent than C1 and C14). This suggests that when both parasite strains have equal chances to infect the host (50:50 concentration), their relative virulence in single infections may point to their competitive success in mixed infections. Similar results have been reported in a rodent malaria host-parasite system [24]. Our study extends these results by showing that even in unequal concentrations P4 produced more or at least as many spores as C1 and C14 during mixed infections with P1/P2, despite its significantly lower spore throughput in single infections. Therefore, the ability of a parasite to transmit under conditions of frequent multiple infections ultimately depends on its competitiveness, and that a parasite’s relative virulence (but not its replication rate) in single infections serves as a good indicator of its competitive ability. Moreover, if more virulent parasite strains are more often better competitors, frequent multiple infections will lead to higher levels of virulence [13].

*Pasteuria ramosa* clones have been found to exhibit strong GxG interactions for infectivity [43]. Some *D. magna* clones exhibit either complete resistance or complete susceptibility to infection that is governed by a simple genetic basis (i.e., one or few loci with dominance; [50]). Although the specificity of attachment to the host esophagus depends on both host and parasite genotypes [51], the specificity of *P. ramosa* proliferation within *D. manga* is poorly understood. It is also unknown whether the number of successful infections (i.e., number of spores attaching to the host esophagus) affects parasite replication rates within the host and the resulting spore load. Single-spore infection trials in the laboratory suggest that even though a single *P. ramosa* spore can cause disease, the likelihood of such an event is extremely low (circa 1 in 700; [43]). Spores that do not penetrate do not seem to be targeted by any innate immunity [52]. It might very well be that if *P. ramosa* spores penetrate the host in small numbers, they are cleared by the host’s innate immune system before they are able to proliferate [53]. Direct interference or apparent competition among different *P. ramosa* clones may also reduce proliferation [54]. Since it is likely that *P. ramosa* isolates consist of more than one clone, some of which may be incompatible with the *D. magna* clone used in this experiment, we conjecture that a combination of proliferation specificity and inter-clone competition may explain why *P. ramosa* clones produced more spores than isolates. In other words, infection by a *P. ramosa* clone would maximize parasite fitness better than infection by a *P. ramosa* isolate. It remains to be determined whether the observed GxG interactions for infectivity also apply to within-host competitiveness and virulence (by examining the expression and evolution of virulence using additional *D. magna* clones).

Our finding that the competitive outcome is largely determined during the first half of the parasite's growth phase may be explained in several ways. First, the replication rates of successful competitors may be considerably higher than those of their counterparts, as evident from spore counts on day 20 post-infection. Second, direct interference or apparent competition might take place very early in the infection process e.g., [53], and clear out or considerably harm less competitive *P. ramosa* clones. Lastly, successful competitors might be able to facultatively upregulate their replication rates upon detection of another genotype within the same host, and thus express higher virulence [55-58]. To provide support to one or more of these conjectures would necessitate monitoring the competitive outcome during the initial growth phase while controlling for the number of successful infections.

Interestingly, the virulence of *P. ramosa* clones was lower than that of isolates. In theory under a scenario of resource competition, kin selection should reduce the increase in virulence per genotype in multiple infections by closely-related competing genotypes [59,60]. However, the relationship between virulence and relatedness depends on the social behavior displayed by the parasites, i.e., prudent exploitation, public goods cooperation or spite [14]. Evidence for reduced overall virulence in coinfections by closely-related parasite strains compared to unrelated strains is scarce [15,16,61]. Under the assumption that more than one *P. ramosa* spore penetrates the host during seven days of exposure, the present study provides additional support for the prediction that high relatedness selects for prudent exploitation and thus low virulence. This is because the difference in virulence between *P. ramosa* clones and isolates could be explained by <100% relatedness of genotypes in isolate infections. This latter statement assumes that a *P. ramosa* isolate consists of more than one *P. ramosa* clone. It remains to be seen whether the increase in overall virulence under multiple infections with potentially unrelated genotypes resulted from increased host exploitation or the inability of the *D. magna* immune system to cope with antigenic diversity [17].

The dose levels used in the present study were chosen to achieve high infection rates (>90%). Infection prevalence in natural populations of *D. magna* varies widely and may reach in certain ponds or years 100% [62-65]. However, it is unknown whether naturally occurring *D. magna* populations are exposed to concentrations of *P. ramosa* spores similar to those administered in our experiment. Lower spore concentrations may decrease the likelihood of multiple infections, and thus alter both within- and between-host dynamics. For example, if multiple infections are rare, less virulent *P. ramosa* clones that produce more transmission stages may be selected over more virulent clones that are less infective and/or produce fewer transmission stages [19]. We do not expect different dose–response relationships for lower levels of infection, in terms of within-host competitiveness, overall virulence and parasite transmission. However, changes in the likelihood of multiple infections will affect the evolution of virulence.

## Conclusions

The main finding of this study is that parasite isolates differ from parasite clones in their virulence and lifetime spore production of an infection. Moreover, parasite isolates/clones differ in their within-host competitiveness and ability to induce host castration. Finally, the relative virulence and relative dose of coinfecting parasite strains strongly affect the competitive outcome. Taken together, our results emphasize the importance of epidemiology as well as of various parasite traits in determining the outcome of within-host competition. Incorporating realistic epidemiological and ecological conditions when testing theoretical models of multiple infections [66], as well as using a wider range of host and parasite genotypes, will enable us to better understand the course of virulence evolution.

## Methods

### Biological system

The host, *Daphnia magna* Straus, is a cyclical parthenogenetic crustacean parasitized by a wide variety of bacterial, microsporidial, oomycetes and fungal parasites [67,68]. The parasite, *Pasteuria ramosa* Metchnikoff 1888, is an endospore-forming, gram-positive bacterium of *Daphnia* with strict horizontal transmission, in which infective stages (i.e., spores) are released from the decaying cadaver of the host [67,69]. It castrates and severely reduces the survival of the host, which rarely produces any offspring after infection [44]. Infections are clearly visible two weeks post-infection, because infected animals have a brownish-reddish color and do not carry eggs. In field populations of *D. magna*, many parasites may coexist in the same pond and multiple infections of host individuals by several *P. ramosa* strains [70,71] or different parasite species [72-74] are often observed.

### Host and parasite collections

We used a single *D. magna* clone (HO2) originally collected from a pond in Hungary, by isolating parthenogenetic eggs from the brood chamber of an uninfected adult female and raising the clonal offspring in isolation under standardized laboratory conditions. In preparation to the experiment we stock-cultured *D. magna* in 400-mL glass beakers, each containing eight individuals with artificial medium [75,76], where they were fed daily with 1.5 × 10^5^ cells mL^-1^ medium of the chemostat-cultured unicellular algae *Scenedesmus gracilis*.

The three *P. ramosa* isolates used in this experiment were obtained either from one infected *D. magna* female (P1: Gaarzerfeld, Germany, 1997; P2: Kains, England, 2002), or from several infected *D. magna* individuals (P4: Heverlee, Belgium, 2003). Isolates are a naturally occurring feature of the *Daphnia*-*Pasteuria* host-parasite system. As such, they are relevant to evolutionary processes in natural populations. These isolates had been used in the laboratory in the past 15 years, and all of them were propagated through the experimental host clone HO2, to obtain enough spore-carrying cadavers to produce sufficient amounts of spore suspensions for the experiment. Two of these isolates (P1 and P4) were also used in a previous study of multiple infections of *D. magna*[19]. The use of laboratory-maintained lines is not unusual in many experimental host-parasite model systems and can be justified by the use of these lines to test mechanistic hypotheses, as we do in our study. The *P. ramosa* clones C1 and C14 were obtained respectively from isolate P5 (Moscow, Russia, 1996) and isolate P3 (Tvärminne, Finland, 2002) via infection by limited dilution (technical details in [43]). These *P. ramosa* clones were also propagated through the experimental host clone HO2. All cadavers were carefully homogenized and spore concentrations were determined using a Thoma counting chamber (depth: 0.02 mm, square width: 0.05 mm).

### Experimental design and setup

We followed a cohort of 1,344 *D. magna* individuals and examined the outcome of single and mixed infections. In total there were 39 treatments, each with 28 replicates, as depicted in Table 3: 20 single infection treatments (three *P. ramosa* isolates and two *P. ramosa* clones, each using four dose levels), 18 mixed infection treatments (combinations of either P1 or P2 with one of P4, C1 and C14, using spore mixtures of 90,000:10,000, 50,000:50,000 and 10,000:90,000 spores), as well as an unexposed control group. In nine of the 18 mixed infection treatments (those with P1), we doubled the number of replicates and used the extra 28 replicates to examine parasite spore production and the competitive outcome on day 20 post-infection. Throughout the experiment and on a daily basis, we monitored *D. magna* survival, release of offspring and the amount of *P. ramosa* spores following the host’s death. We defined virulence as time-to-host-death-since-exposure (i.e., host longevity). We chose this definition, instead of “reduction in host fitness following infection”, because the trade-off model is based on “parasite-induced host mortality” being the right definition for the virulence of horizontally-transmitted parasites [77]. Model predictions using expected host longevity may differ from those obtained using host mortality rate [78], yet in our one-generation study these effects are most likely negligible. Host fitness was defined as the lifetime number of offspring produced. Parasite fitness was estimated from the number of spores at the time of host death, which is equal to the lifetime spore production of an infection.

**Table 3:** Overview of the treatments in the experiment

**Treatment**	**Type of infection**	**Infection dose**	**Abbreviations of combinations used**
PX-10	Single	10,000 spores of PX	P1-10, P2-10, P4-10, C1-10, C14-10
PX-50	Single	50,000 spores of PX	P1-50, P2-50, P4-50, C1-50, C14-50
PX-90	Single	90,000 spores of PX	P1-90, P2-90, P4-90, C1-90, C14-90
PX-100	Single	100,000 spores of PX	P1-100, P2-100, P4-100, C1-100, C14-100
PX-10 + PY-90	Mixed	10,000 spores of PX and 90,000 spores of PY	P1-10 + P4-90, P1-10 + C1-90,
			P1-10 + C14-90, P2-10 + P4-90,
			P2-10 + C1-90, P2-10 + C14-90
PX-50 + PY-50	Mixed	50,000 spores of PX and 50,000 spores of PY	P1-50 + P4-50, P1-50 + C1-50,
			P1-50 + C14-50, P2-50 + P4-50,
			P2-50 + C1-50, P2-50 + C14-50
PX-90 + PY-10	Mixed	90,000 spores of PX and 10,000 spores of PY	P1-90 + P4-10, P1-90 + C1-10,
			P1-90 + C14-10, P2-90 + P4-10,
			P2-90 + C1-10, P2-90 + C14-10
Control	None	None	Control

In single infections, PX stands for either one of the three *P. ramosa* isolates P1, P2 and P4, or one of the two *P. ramosa* clones C1 and C14. In mixed infections, PX stands for either P1 or P2, and PY stands for one of P4, C1 and C14.

We used offspring of the third generation of the HO2 isofemale line to minimize maternal effects. To start the experiment we separated newborns from the *D. magna* clone line (0–24 h old) into four 400-mL beakers and fed them daily with 1.5 × 10^5^ algae cells mL^-1^ medium. On day four we placed single females of *D. magna* in 100-mL jars, filled with 20 mL of artificial medium, and initially fed them 2 × 10^6^ algae cells per animal per day. The infection treatment was performed on day five. A week later, on day 12, we replaced the medium of all animals with 100 mL of fresh medium and thereafter medium was replaced on a weekly basis. To accommodate the growing food demands of the growing animals, on days 9, 15, 18, 22, 27, 30 and 37 we increased the daily food level for all individuals to 3 × 10^6^, 5 × 10^6^, 6 × 10^6^, 7 × 10^6^, 8 × 10^6^, 9 × 10^6^ and 10 × 10^6^ algae cells per day, respectively.

The temperature was 20 ± 0.5°C and the light:dark cycle was 16h:8h. All treatments were randomly distributed across the shelves of two incubators and their position was rearranged frequently to avoid position effects. Offspring counts and dead animals were recorded daily. Animals that had died after day 16 (since birth) were dissected and checked for disease using phase contrast microscopy (300-600×). Animals that had died earlier could not be reliably scored for infection and were thus excluded from the analyses. The experiment was terminated after all animals had died. The dead *D. magna* were frozen in 0.1 mL of medium at −20°C for subsequent parasite spore counting with a haemocytometer.

### Genetic analyses

To trace the relative success of *P. ramosa* isolates and clones during mixed infections, we used variable number of tandem repeats (VNTR) markers. We used the previously developed primers Pr1, Pr2 and Pr3 (for details, see [70] and Table 2 in [19]). These primers allow distinguishing between *P. ramosa* isolates P1 and P3/P4/P5, and between isolates P2 and P3/P4/P5. Because *P. ramosa* clones C1 and C14 were derived from isolates P5 and P3, respectively, the same primers can be used to distinguish between isolate P1 and clones C1/C14, and between isolate P2 and clones C1/C14. The protocol employed resembles the one used by [19] with the following changes. Spore solutions were suspended in 300 μL water and 30 μL proteinase K (20 mg/mL). We added approximately 160 mg of 0.1 mm zirconia beads, subjected them to beating for 20 s at full speed, and incubated them at 56°C for 30 min. We then spinned down beads at 5000 g for 30 s, transferred supernatant to a new tube and continued with peqGOLD Tissue DNA Mini Kit (Peqlab, Erlangen, Germany). The relative intensity of the peaks of the *P. ramosa* isolate/clone specific microsatellite markers were analyzed with AB3130xl Sequencer (Applied Biosystems, Foster City, USA) and interpreted as the relative proportion of spores of different *P. ramosa* isolates/clones in an individual *D. magna* as described in [19]. Spore counts for each *P. ramosa* isolate/clone in mixed infections were derived by multiplying the abovementioned relative proportion by the total number of spores produced following infection.

### Statistical analyses

All statistical tests were done using SPSS for Windows release 19.0.0.1 (SPSS Inc. 2010). The effects on virulence, host and parasite fitness were investigated using general linear models (GLM). Because time-to-host-death was normally distributed and the experiment ended only after all hosts had died, there was no need for censoring data and using specific survival analysis procedures. When necessary, parasite spore production and host offspring counts were square-root-transformed to meet the normality and homoscedasticity assumptions. In GLM procedures dose level and parasite isolate/clone were considered fixed factors. Dose level was only used to compare single infection treatments with spore mixtures of 10,000, 50,000, 90,000 and 100,000 spores (see Table 2). The dichotomous variable parasite isolate/clone was used to compare the virulence and spore production of *P. ramosa* isolates vs. clones. Thereafter, contrasts were used to test specific hypotheses in subsets of the total dataset.

## Competing interests

The authors declare that they have no competing interests.

## Authors’ contributions

FBA conceived and designed the study, carried out the infection assays, performed the statistical analysis and drafted the manuscript. JR carried out the molecular genetic work and helped to draft the manuscript. Both authors read and approved the final manuscript.
